# Effect of Age on Innate and Adaptive Immunity in Hospitalized COVID-19 Patients

**DOI:** 10.3390/jcm10204798

**Published:** 2021-10-19

**Authors:** Lamin B. Cham, Marie Høst Pahus, Kristoffer Grønhøj, Rikke Olesen, Hien Ngo, Ida Monrad, Mads Kjolby, Martin Tolstrup, Jesper Damsgaard Gunst, Ole S. Søgaard

**Affiliations:** 1 Department of Infectious Diseases, Aarhus University Hospital, 8200 Aarhus, Denmark; laminbcham@clin.au.dk (L.B.C.); mariehp@clin.au.dk (M.H.P.); kristoffergroenhoej@gmail.com (K.G.); RIKKOL@rm.dk (R.O.); hienngo@clin.au.dk (H.N.); idprha@rm.dk (I.M.); marttols@rm.dk (M.T.); JESDAM@rm.dk (J.D.G.); 2 Department of Clinical Medicine, Aarhus University, 8200 Aarhus, Denmark; mads@dandrite.au.dk; 3 Department of Clinical Pharmacology, Aarhus University Hospital, 8200 Aarhus, Denmark; 4 Steno Diabetes Center Aarhus, Aarhus University Hospital, 8200 Aarhus, Denmark

**Keywords:** SARS-CoV-2, COVID-19, age, monocytes, DCs, NK cells, T cells, clinical recovery

## Abstract

An effective but balanced cellular and inflammatory immune response may limit the severity of coronavirus disease (COVID-19), whereas uncontrolled inflammation leads to disease progression. Older age is associated with higher risk of COVID-19 and a worse outcome, but the underlying immunological mechanisms for this age-related difference are not clear. We investigated the impact of age on viral replication, inflammation, and innate and adaptive cellular immune responses in 205 hospitalized COVID-19 patients. During the early symptomatic phase of COVID-19, we found that patients above 65 years had significantly higher viral load, higher levels of proinflammatory markers, and inadequate mobilization and activation of monocytes, dendritic cells, natural killer cells, and CD8 T cells compared to those below 65 years. Our study points toward age-related deficiencies in the innate immune cellular response to SARS-CoV-2 as a potential cause of poorly controlled viral replication and inflammation during the early symptom phase and subsequent disease progression.

## 1. Introduction

The outbreak and spread of severe acute respiratory syndrome coronavirus 2 (SARS-CoV-2) has posed one of the most serious global health and socioeconomic crises of our time [1,2]. The pandemic has affected every nation across the globe [3], infecting over 235 million people and claiming over 4.8 million lives estimated by the World Health Organization (WHO). Unlike the previous coronavirus outbreaks, recent globalization coupled with the rapid SARS-CoV-2 person to person transmission are key factors for the highly efficient spread across nations [4,5,6,7,8].

SARS-CoV-2 is the etiological cause of coronavirus disease 2019 (COVID-19) which is characterized by an acute respiratory disease ranging from asymptomatic infection to severe pneumonia and acute respiratory failure. Upon infection, it usually takes between 3 and 7 days before onset of the first symptoms such as fever, dry cough, tiredness, sore throat, etc. [9,10,11,12]. The level of SARS-CoV-2 replication, risk of infection and transmission, and disease severity depend on host immunological and inflammatory responses and other biochemical, molecular, and physiological factors [13,14,15].

Understanding virus–host interactions at immunological and molecular levels is important for the development of antivirals and vaccines in the control of any outbreak [16]. Host suppression and control of SARS-CoV-2 replication, like any other viral infection, requires effective innate and adaptive immune responses. Recent SARS-CoV-2 findings support the strong association of innate immunity in determining disease severity [17,18,19,20,21]. Interestingly, SARS-CoV-2 employs several strategies to inhibit innate immunity such as suppressing type 1 interferon responses as well as broad functional impairment of the function of dendritic cells (DC), natural killer (NK) cells, monocytes, and macrophages. Upon infection, SARS-CoV-2 significantly suppress antigen presentation process by downregulating the costimulatory molecules (CD86 and CD40) on antigen-presenting cells [22,23,24,25]. This functional impairment of antigen-presenting cells compromises T cell maturation and activation, thereby reducing the antigen-specific T cell responses against SARS-CoV-2 [26]. Reports have also shown that SARS-CoV-2 induces T cell apoptosis by promoting extrinsic and intrinsic apoptosis pathways and promoting early CD8 T cell exhaustion [27,28]. In addition to the suppression of cellular immunity, SARS-CoV-2 infection induces elevated levels of proinflammatory cytokines and chemokines (e.g., IL-6, TNF-α, IP-10, etc.), which have been associated with higher risk of disease progression [29,30].

Aging is a key physiological factor that can influence disease onset and progression [31,32,33,34]. Similar to cancer, degenerative disorders, and other noninfectious diseases, age is also a well-described factor in infectious diseases; for instance, older HIV-infected individuals have twice the risk of progressing to AIDS compared to younger patients [35,36]. Mechanistically, the pathophysiological phenomena underlying the influence of age is the physiological decline in immune competence, also known as immunosenescence [37]. The cellular immune and inflammatory response to infections is a double-edged sword that can mediate a protective immunity or lead to severe immunopathology due to an imbalance and inappropriate production of cytokines and chemokines. Immunosenescence and dysregulated inflammation in elderly patients may increase the risk of disease progression and poor clinical outcomes in COVID-19 [38,39,40,41]. During the COVID-19 pandemic, countless devasting and difficult-to-control disease outbreaks in nursing homes have been reported [42,43,44,45,46]. Reasons why the virus spreads so effectively in nursing homes compared to school classes or workplaces are multifactorial, but it is likely to include a biological component. We therefore investigated how age influences SARS-CoV-2 replication, innate and adaptive immune responses, and clinical outcomes among newly admitted COVID-19 patients.

## 2. Materials and Methods

### 2.1. Study Population

The study population (205 patients) was derived from a cohort of PCR-confirmed hospitalized COVID-19 patients who were enrolled in a clinical trial [47]. Individuals for whom there were no cryopreserved peripheral blood mononuclear cells (PBMCs) at baseline, were pregnant, were breastfeeding, or had serum total bilirubin × 3 above the upper limit of normal were excluded from the study. PBMCs, plasma, nasopharyngeal swabs, and clinical data such days of symptom onset were collected upon admission.

### 2.2. Peripheral Blood Mononuclear Cell (PBMC) Isolation

PBMCs from patients were isolated from fresh blood samples using Ficoll-Paque density gradient centrifugation after blood collection. The majority of purified PBMCs were used for immune cell phenotyping, whereas plasma samples were used for biochemistry, IgG, and cytokine profiling.

### 2.3. Quantification of SARS-CoV-2

Nasopharyngeal swabs were collected with Copan ESwabTM (1 or 2 mL medium) from participants on day 1 and were subsequently analyzed using digital droplet PCR (ddPCR) to quantify viral load. RNA was extracted from at least 280 mL swab medium using QIAamp Viral RNA Mini Kit (Qiagen) following the manufacturer instructions and the resulting RNA eluted in 70 uL AVE buffer. To quantify SARS CoV-2 in the swabs using ddPCR, primers/probe targeting the nucleocapsid in SARS CoV-2 was used; N1 Forward: 5′-GACCCCAAAATCAGCGAAAT-3′, N1 Reverse: 5′-TCTGGTTACTGCCAGTTGAATCTG-3′ and N1 probe: 5′-6FAM-ACCCCGCATTACGTTTGGTGGACC-3′. RPP30 was used as internal control; Forward: 5′-GATTTGGACCTGCGAGCG-3′, Reverse: 5′-GCGGCTGTCTCCACAAGT-3′ and probe: 5′-6FAM-CTGACCTGAAGGCTCT-3′. The ddPCR reactions consisted of the One-Step RT ddPCR Adv. kit (Bio-Rad) with the recommended concentrations of reagents and 10 mL RNA. Droplets were generated using an Automated Droplet Generator (Bio-Rad, Copenhagen, Denmark) and the following PCR program was used (25 °C for 3 min, 50 °C for 60 min, 95 °C for 10 min, then 40 cycles of 95 °C for 30 s and 55 °C for 1 min, then 98 °C for 10 min and 12 °C) prior to droplet analysis in a QX200 Droplet Reader (Bio-Rad, Copenhagen, Denmark). N1 reactions were run in duplicates and RPP30 in singlets. The limit of the blank was calculated as 168, 8 copies/mL from 36 non-template controls, and the lower limit of quantification was set to 200 copies/mL. For samples with high viral load, the RNA was diluted until quantification by ddPCR was possible.

### 2.4. Viral Sequence

Patient samples were sequenced by the Danish COVID-19 Genome Consortium (DCGC) at Aarhus University Hospital (https://www.covid19genomics.dk, accessed on 10 September 2021). The SARS-CoV-2 genome was amplified using QIAseq SARS-CoV-2 Primer Panel (Qiagen 333896) and, subsequently, libraries were made with QIAseq FX DNA library UDI kit and performed according to manufacturer’s protocol. The libraries were sequenced (150 bp paired-end) on Illumina NextSeq 500 (Illuminia, Cambridge, UK).

### 2.5. Cell Surface Staining

Cryopreserved PBMCs were thawed and stained with viability dye (Near-IR) for 20 min at 4 °C. Nonspecific binding was blocked, and cells were stained with cell surface stain antibodies (in four different flow panels, e.g., DC, Monocytes, NK cell, and T cell panel) for 30 min at 4 °C. Flow cytometry was performed using a BD LSRFortessa™ X-20 Cell Analyzer (BD bioscience, New Jersey, NJ, USA), and analysis of all FACS files was performed in flow-Jo. Details of the list of antibodies, source, and identifier are shown in Supplementary Table S2.

### 2.6. Inflammatory Cytokines

Cytokine (IL-2, IL-6, IFN-γ, IP-10, TNF-α, etc.) plasma levels were measured using V-PLEX Custom Human Cytokine 54-plex kits for cytokines that were purchased from Meso Scale Discovery (MSD). Assays were performed according to manufacturer’s protocol with overnight incubation of the diluted samples and standards at 4 °C. The electrochemiluminescence signal (ECL) were detected by MESO QuickPlex SQ 120 plate reader (MSD, New Jersey, NJ, USA) and analyzed with Discovery Workbench Software (v4·0, MSD, New Jersey, NJ, USA).

### 2.7. Biochemistry Assay

CRP and ferritin levels were measured at the clinical biochemistry laboratory of the Aarhus University Hospital. Several biochemical markers including CRP and ferritin levels were analyzed from patient serum samples using the automated ADVIA^®^ Chemistry XPT-system (Siemens, Ballerup, Denmark). Assay was performed according to manufacturer’s protocol and a standard curve was used for quantitation.

### 2.8. Serological Responses

IgG antibodies were measured in serum samples using the MSD COVID-19 Coronavirus Panel 1 (Cat. No. K15362U-2, MesoScale Discovery, Rockville, MD, USA), a solid phase multiplex immunoassay, with 10 precoated antigen spots in a 96-well format, with an electrochemiluminescence-based detection system. The SARS-CoV-2 antigen used was the SARS-CoV-2 spike. Bovine serum albumin (BSA) served as negative control spot. Nonspecific antibody binding was blocked using MSD Blocker A. CamoCO-19 patient serum samples and control samples were diluted 1:278 in MSD Diluent 100. Reference Standard 1 was used as assay calibrator. Serology controls 1.1, 1.2, and 1.3 were used for internal control of assay performance. After sample incubation, bound IgG was detected by incubation with MSD SULFO-TAG Anti-Human IgG Antibody and subsequently measured on a MESO QuickPlex SQ 120 Reader (MSD, New Jersey, NJ, USA) after addition of GOLD Read Buffer B. IgG concentrations were calculated in arbitrary units (AU)/mL according to the Reference Standard 1 assay calibrator. Samples measurements above detection range were assigned the upper value of the calibration range.

## 3. Quantification and Statistical Analysis

### Statistical Analysis

The data are shown as either mean ± SEM or median (interquartile range (IQR)). In Table 1, we used Fisher’s exact test on *n* (%) and the Mann-Whitney *U*-test on median (IQR) to compare the two groups. Student’s *t*-test was used to detect statistically significant differences between groups at each time point.

A planned analysis using *t*-test at each timepoint (without correcting for multiple tests) was used, because it was hypothesized that the influence of age would be different at different stages of COVID-19.

The level of statistical significance was set at *p* < 0.05. Graphs were prepared with GraphPad Prism 8 (GraphPad Software, La Jolla, CA, USA).

## 4. Results

### 4.1. Baseline Characteristics of the COVID-19 Study Population

Clinical characteristics at hospital admission of the 205 COVID-19 patients are shown in (Table 1). We divided the patients into two groups: below 65 years (116 patients) or above 65 years (89 patients) of age. We observed a higher median time from symptom onset to hospital admission in the <65 years group (9 days) compared to the >65 years group (7 days). The younger patients tended to have more symptomatic manifestations such as coughing, dyspnea, fatigue, and headache on admission. The older patients on the other hand had more coexisting conditions such as COPD, coronary heart disease, hypertension, malignancy, and type 2 diabetes. Observational studies indicated that older compared to younger patients with cardiovascular disease or risk factors are more vulnerable and increased morbidity and mortality from COVID-19 [48,49,50]. In addition to the increased level of comorbidity, we also observed a higher percentage of individuals requiring nasal oxygen therapy upon admission among >65 years patients.

### 4.2. Age Is Associated with Increased SARS-CoV-2 Viral Load, Plasma Inflammation Markers and Delayed Clinical Recovery

**Figure 1:**
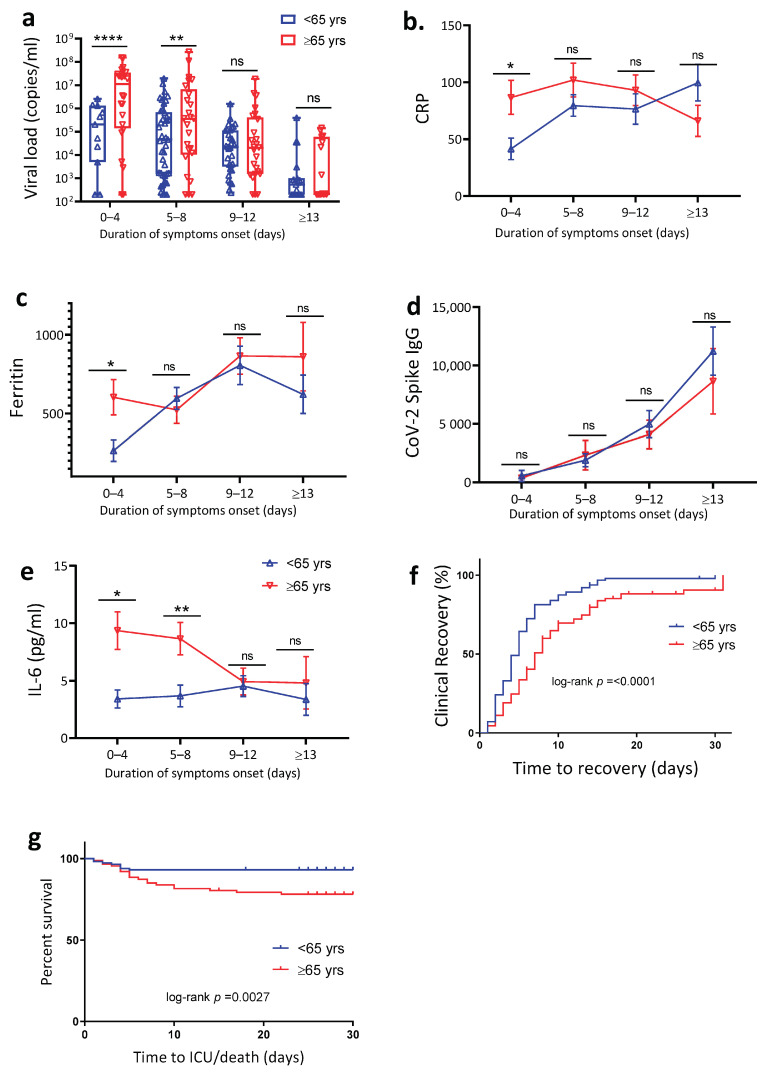
Age is associated with increased SARS-CoV-2 viral load, inflammatory response, and delayed clinical recovery. Oropharyngeal swabs and serum sample of SARS-CoV-2 patients were analyzed for viral titer, biochemistry, IgG, and cytokine level. (**a**) Quantification of patients SARS-CoV-2 viral load by droplet PCR (ddPCR) from oropharyngeal swabs (*n* = 193). (**b**) Serum CRP and (**c**) serum ferritin level were determined using clinical-based biochemistry assay (*n* = 193). (**d**) Levels of SARS-CoV-2-specific IgG in serum (*n* = 193). (**e**) Cytokine profiling of serum IL-6 level (*n* = 89). Kaplan-Meier estimate of (**f**) time to clinical recovery and (**g**) time to intensive care unit or death. The data are shown as mean ± SEM. The statistical comparison of below 65 years (<65 years) vs. above 65 years (≥65 years) was performed using Student’s *t*-test (* *p* < 0.05, ** *p* < 0.01, **** *p* < 0.0001).

To account for differences in duration of COVID-19, we further subcategorized the study population according to time from symptom onset to admission, i.e., number of days between the first reported symptom until the day of hospital admission 0 to 4 days (38 patients), 5 to 8 days (54 patients), 9 to 12 days (67 patients), and ≥13 days (44 patients). Using a ddPCR assay, we quantified SARS-CoV-2 viral load from oropharyngeal swabs and found that COVID-19 patients >65 years compared to those <65 years had significantly higher viral load in the first eight days of symptom onset (Figure 1a). To evaluate whether the higher viral load in the older patients could have resulted from infection with more virulent SARS-CoV-2 strains, we performed full viral genome sequencing of extracted RNA from oropharyngeal swabs (from 101 patients). The sequencing analysis identified strains that were representative of those reported to be circulating in Denmark at the time of sample collection. Only two B.1.1.7 isolates were identified, one in each group. The predominant strain B.1.177 was present in 25 (50.0%) and 14 (27.4%) in the <65 years group and >65 years group, respectively (Supplementary Table S1). Further analyses of biochemistry plasma inflammatory markers showed that >65 years patients had increased CRP (Figure 1b), increased ferritin (Figure 1c), and increased IL-6 levels (Figure 1e) in the early phase of symptom onset. In contrast, we found no significant differences in total SARS-CoV-2 spike IgG between to two age groups (Figure 1d) suggesting that time of infection correlated well with the self-reported symptom duration. Finally, time to clinical recovery (Figure 1f) was longer and disease progression to intensive care unit admission or death higher among the >65 years compared to the <65 years group (Figure 1g). Collectively, these findings suggest a strong association between aging, SARS-CoV-2 viral load, inflammation, and disease outcome.

### 4.3. Elderly Patients Exhibited Reduced Monocyte Activation and Function

**Figure 2:**
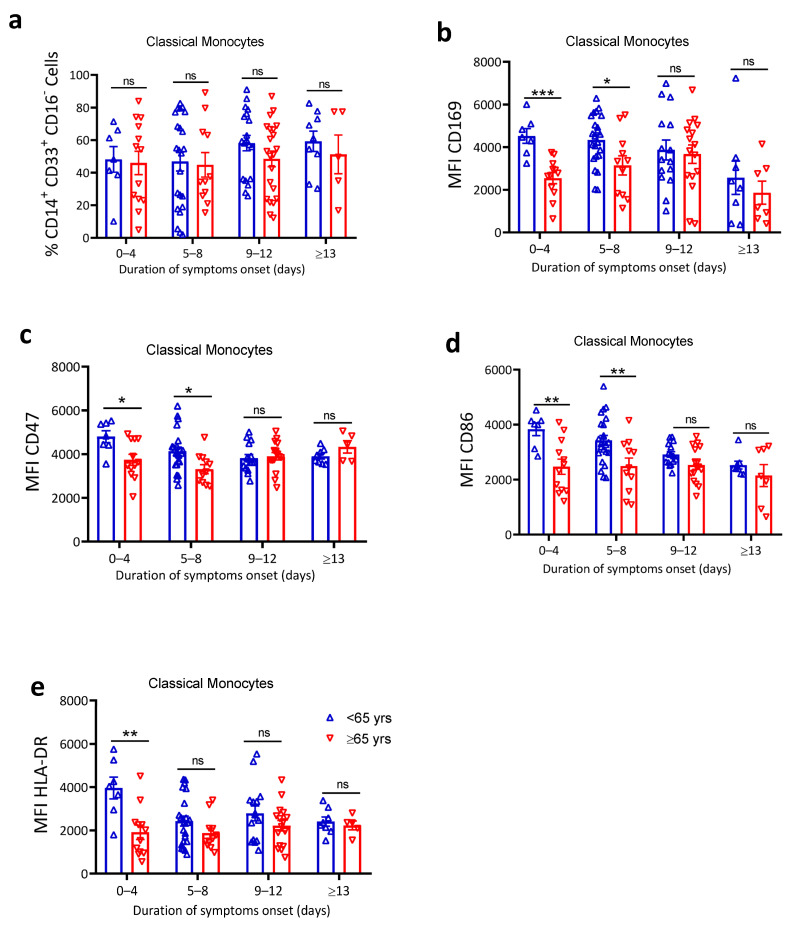
Elderly patients exhibited reduced monocyte activation and function. Flow cytometry analysis was performed on thawed PBMC from SARS-CoV-2 patients after an indicated timepoint of duration of symptom onset. Classical monocytes frequency and activation was analyzed. (**a**) Percentage of classical monocytes (% CD14^+^CD33^+^CD16^−^ monocytes), mean fluorescence intensity (MFI) of (**b**) CD169, (**c**) CD47, (**d**) CD86, and (**e**) HLA-DR (*n* = 101). The data are shown as mean ± SEM. The statistical comparison of below 65 years (<65 years) vs. above 65 years (≥65 years) was performed using Student’s *t*-test (ns: not significant, * *p* <0.05, ** *p* < 0.01, *** *p* < 0.001).

To understand how the function of one of the key innate immune cells in the initial response to viral infections may be impacted by age, we performed flow cytometry on cryopreserved peripheral blood nuclear cells (PBMC). Monocyte proportion, activation, and function were analyzed using the gating strategy in (Supplementary Figure S1a). We observed no significant difference in the percentage of monocytes in PBMCs (Supplementary Figure S1b) or in the proportion of classical monocytes out of total monocytes (Figure 2a) between the two age groups. We further looked at CD169, a well-known activation marker of early innate immune cells [51,52], and CD47, a widely recognized antiphagocytic molecule and one of the early interferon-stimulated molecules [53]. The classical monocytes showed significantly higher expression of both CD169 (Figure 2b) and CD47 (Figure 2c) in the first eight days of symptom onset among <65 years compared to >65 years patients. In addition, the intermediate monocytes also displayed increased CD169 and CD86 expression (Supplementary Figure S1c,d) among younger compared to older patients during the early phase of symptom onset. Next, we evaluated the expression of costimulatory molecules CD86 and HLA-DR to investigate whether monocyte capacity to engage T cells was also impacted by age. Our results indicated that elderly patients exhibit significantly reduced expression of both CD86 (Figure 2d) and HLA-DR (Figure 2e) in the first eight days of symptom onset. In addition to the decline monocyte activation in elderly patients, we observed a slightly increased serum proinflammatory IP-10 levels (Supplementary Figure S1e) in older patients, which is reported to be a biomarker associated with COVID-19 severity [54]. Thus, we conclude that patients >65 years have reduced monocytes activation, reduced monocyte antigen presentation capacity, and increased IP-10 cytokine levels in the early phase of symptomatic COVID-19.

### 4.4. Dendritic Cells Impairment in Patients above 65 Years

**Figure 3:**
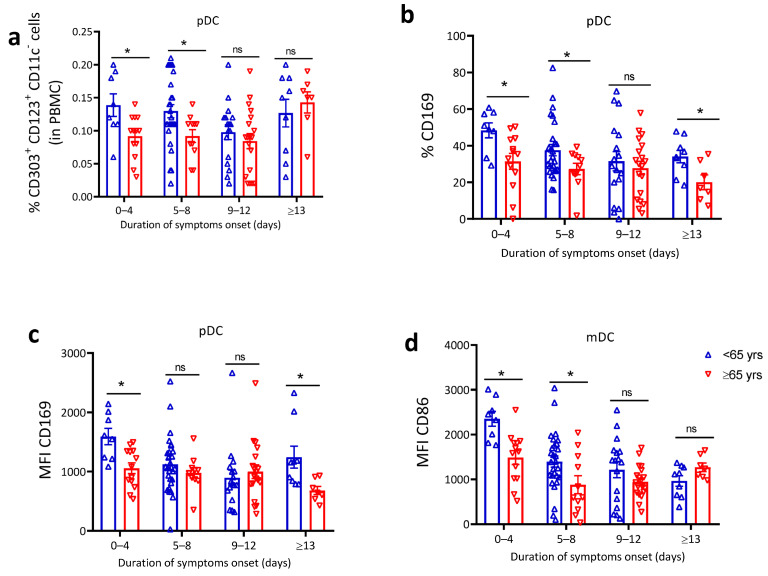
Dendritic cell impairment in patients above 65 years. Analysis of dendritic cell proportion and cell surface activation in PBMC from SARS-CoV-2 patients after indicated duration of symptom onset. (**a**) Percentage of pDC (% CD303^+^CD123^+^CD11c^−^ DCs). (**b**) Percentage of CD169 expressing pDCs. Mean fluorescence intensity (MFI) of (**c**) CD169 and (**d**) CD86 (*n* = 117). The data are shown as mean ± SEM. The statistical comparison of below 65 years (<65 years) vs. above 65 years (≥65 years) was performed using Student’s *t*-test (ns: not significant * *p* < 0.05).

DCs are immune regulatory cells reported to be crucial for the outcome of COVID-19 [55] due to their pivotal role in initiating the innate inflammatory response as well as in priming and activation of the adaptive immune response [56]. We therefore next evaluated how age may affect DC homeostasis and function. Plasmacytoid dendritic cell (pDC) and myeloid dendritic cell (mDC) flow analyses (gating strategy shown in Supplementary Figure S2a) revealed a significantly higher frequency of circulating pDCs in the early phase of symptom onset among patients <65 years compared to >65 years (Figure 3a). In addition, younger COVID-19 patients had higher proportions of CD169 expressing pDC (Figure 3b) and higher CD169 MFI as indicator of enhanced pDC activation (Figure 3c). In addition to the increased pDC activation, mDC from <65 years patients had higher expression of CD86 indicating enhanced capacity for antigen cross-presentation and priming of T cells (Figure 3d). Collectively, these results demonstrate lower DC activation and diminished antigen cross-presentation capacity during the early phase of COVID-19 in older compared to younger patients.

### 4.5. Blunted Natural Killer Cell Activation and Lower IL-2 Levels in Older Patients

**Figure 4:**
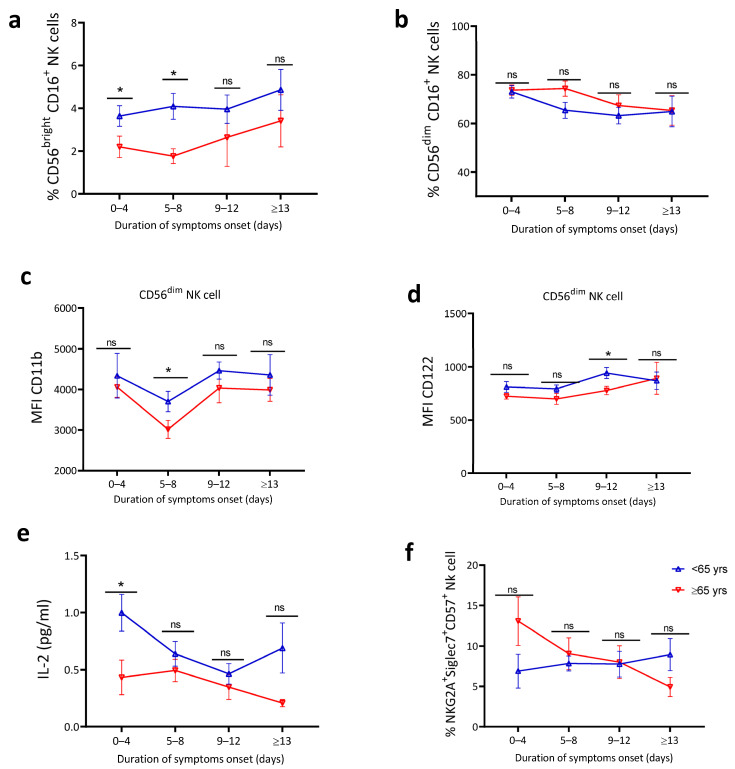
Increased natural killer cell activation and IL-2 levels in younger patients. Natural killer cell response and cytokine level analysis from PBMC and serum, respectively. (**a**) Percentage of CD56^bright^ NK cells. (**b**) Percentage of CD56^dim^ NK cells. Mean fluorescence intensity (MFI) of (**c**) CD11b of CD56^bright^ NK cells, (**d**) CD122 of CD56^dim^ NK cells, (**e**) serum IL-2 level, and (**f**) percentage of NKG2a^+^Siglec7^+^ NK cells (*n* = 83). The data are shown as mean ± SEM. The statistical comparison of below 65 years (<65 years) vs. above 65 years (≥65 years) was performed using Student’s *t*-test (ns: not significant, * *p* < 0.05).

Natural killer (NK) cells have been reported to be important for in the early response to respiratory tract infections [57,58]. Similar to other innate immune cells, we profiled NK cells using flow cytometry to understand the potential impact of age on NK cell activation in COVID-19 patients. The gating strategy for NK cells is shown in Supplementary Figure S2b. Subset analyses of the NK cell department demonstrated that COVID-19 patients <65 years exhibited higher percentages of CD56^bright^ NK cells (Figure 4a) characterized as potent cytokine-producing NK cells, whereas proportions of potent cytotoxic CD56^dim^ NK cells were unchanged (Figure 4b) in younger compared to older patients in the early phase of symptom onset. Younger patients had slightly higher CD11b expression on CD56^bright^ NK cells (Figure 4c) and CD122 on CD56^dim^ NK cells (Figure 4d) compared to elderly patients. Interleukine-2 (IL-2) is an immune stimulatory cytokine that can be produced by NK cells and, in turn, enhance NK and T cell proliferation, activation, and function [59]. Cytokine profiling shows increased IL-2 serum levels *p* = 0.0314 (Figure 4e) in patients <65 years compared to >65 years. We found no significant difference in NK cell inhibitory and/or exhaustion markers as shown in the percentage of NKG2a^+^Siglec^+^ CD57^+^ NK cells (Figure 4f). We conclude that the observed higher levels of NK cell activation, cytokine-producing capacity, and secreted IL-2 among the <65 years group were consistent with the observations in monocyte and DC subsets implying impaired innate immune engagement among older compared to younger patients in the early phase of COVID-19.

### 4.6. Reduced T Cell Activation in Older COVID-19 Patients

**Figure 5:**
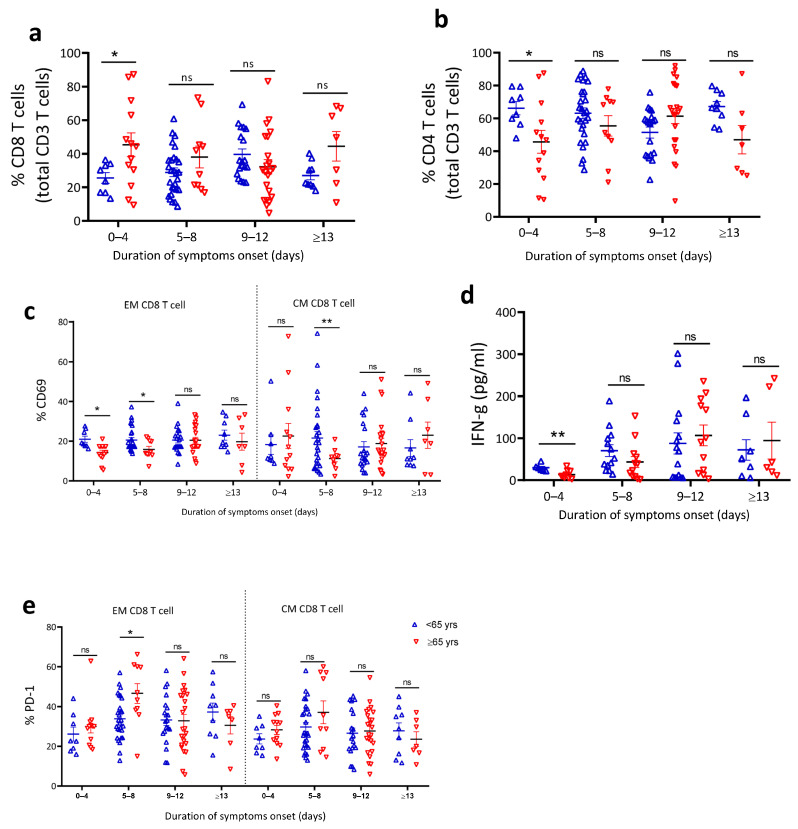
Reduced T cell activation in older COVID-19 patients. Cell surface staining of T cells response and cytokine level from PBMC and serum, respectively. (**a**) Percentage of CD8 T cells and (**b**) percentage of CD4 T cells from total CD3 T cells. (**c**) Percentage of CD69 expression on effector and memory CD8 T cells, (**d**) serum IFN-γ level, and (**e**) percentage of PD-1 expressing effector and memory CD8 T cells (*n* = 117). The data are shown as mean ± SEM. The statistical comparison of below 65 years (<65 years) vs. above 65 years (≥65 years) was performed using Student’s *t*-test (ns: not significant, * *p* < 0.05, ** *p* < 0.01).

The long-term suppression and recovery from SARS-CoV-2 infection depend on robust T cell immunity [60,61]. To compliment the profiling of innate immune cells, we evaluated T cell activation and phenotype characteristics among our patients (gating strategy shown in Supplementary Figure S3a). The analysis revealed that the relative proportions of CD8/CD4 were different between the young and old with <65 having greater proportions of CD4 and less CD8 compared to the old (Figure 5a,b). Both T cells subsets were further subgrouped into naïve, terminally differentiated (TD), effector memory (EM), and central memory (CM), as shown in Supplementary Figure S3b,c. Activation of CD8 effector memory and central memory T cell were lower based on CD69 expression (Figure 5c) in older COVID-19 patients in the early phase of symptom onset. Significantly reduced plasma IFN-γ (Figure 5d) and moderately increased TNF-α levels (Supplementary Figure S3d) were observed among older compared to younger patients at 0–4 days of symptom onset. Finally, following reports that SARS-CoV-2 induces T cell apoptosis and early CD8 T cell exhaustion [27,28,62], we investigated whether age is a determining factor of T cells exhaustion. We found higher proportions of exhausted PD-1 (Figure 5e) positive effector memory and central memory CD8 T cells in older compared to younger COVID-19 patients in the early phase of symptom onset. We conclude that older age was associated with abrogated CD8 T cell activation and early CD8 T cell exhaustion.

## 5. Discussion

In the present study, we combined detailed phenotypic characterization of the major immune cell subsets with quantitative viral load measurements, SARS-CoV-2 antibody measurements, cytokine profiling, and other proinflammatory markers. We interrogated age as determining factor in the interplay between cellular and inflammatory response, SARS-CoV-2 replication, COVID-19 progression, and time to clinical recovery. Collectively, our results showed that aging perturbs the balance in the cellular innate immune and proinflammatory response during SARS-CoV-2 infection and that older age was strongly associated with impairment of both innate and adaptive immunity in COVID-19.

The question that our study addressed was how age impacts the activation and mobilization of the early immune response following SARS-CoV-2 infection. For a host immune system to successfully fight and clear a new invading pathogen, the response needs to be balanced and involve effective innate activation and rapid priming of adaptive immune cells. Unbalanced cellular and inflammatory immune responses can lead to immunopathology and hyperinflammation, but an impaired immune response can also lead to poor viral control and viral persistence [56,63,64]. We now know that hyperinflammation is a hallmark in the development of severe and fatal COVID-19 [61,62,63], and it is also clear that age is a key contributing risk factor for disease progression. In line with this evidence, we found that the level of several soluble proinflammatory markers were increased (e.g., CRP, ferritin, IP-10, and IL-6) in the >65 years compared to <65 years COVID-19 patients. During the early symptom phase, patients >65 years had up 1.5 log_10_ higher viral load in the oropharyngeal swabs compared to <65 years patients. As has been reported in other studies, we also observed a higher risk of COVID-19 progression and delayed time to clinical recovery among >65 years patients [54,63,64,65]. These findings demonstrated age as crucial risk factor for hyperinflammation, poor virological control, and increased risk of disease progression.

To understand the potential underlying immunopathology, we focused our investigations on the innate immune system. First, we evaluated monocytes and other immune subsets phenotype from patients’ PBMCs. Our phenotypic characterization of monocytes revealed no significant age difference in the frequency of total monocytes, nor did we observe any increase in the proportion of classical monocytes or other monocytes subsets. Determining the relevance of monocytes activation in antiviral immunity, we evaluated monocytes early activation markers (CD169 and CD47). Increased CD169 expression on monocytes is directly proportional to IFN-I plasma levels (52), although we have not performed any assay to determine IFN-1 levels among these patients, but our results showed decreased CD169 and decreased CD47 expression among >65 years patients. Considering the role of monocytes and IFN-I in suppression of early SARS-Cov-2 replication [66], the observed decreased monocytes activation in >65 years patients may suggest less control of SARS-CoV-2 replication in the first phase of the disease, which is in line with our observation of higher viral loads in older patients. In addition to mediating a direct antiviral effect, monocytes also function as antigen-presenting cells. Of note, SARS-CoV-2 has several immune invasion strategies, and one of these strategies includes the downregulation of antigen presentation molecules such as MHC-I and T cell costimulatory ligands [67]. Indeed, we found decreased expression of costimulatory (CD86) molecule and downregulation of MHC-II surface receptor (HLA-DR) on monocytes. Thus, aging is strongly associated with impaired monocyte antiviral function as well as reduced ability to cross-activate T cells in COVID-19.

DCs are known as master regulators of the early response to pathogens, and they act as messengers between the innate and adaptive immune responses. A key observation of our study is the association of increasing age with a decline in DC number and function. This could potentially contribute to low levels of IFN-I and reduced activation of other immune subset (e.g., NK and CD8 T cells) in our COVID-19 patients. A well-timed and robust pDC mediated IFN-I response induce potent suppression of viral replication in the early stage of SARS-CoV-2 infection [26]. Profiling of dendritic cells revealed a significant reduction in pDC numbers and reduced surface expression of the CD169 early activation marker on pDC in >65 years compared to <65 years patients. mDCs are the most potent antigen-presenting cells. In our analysis, mDCs showed a similar trend to pDCs with downregulation of surface expression of CD86 among >65 years patients. The reduced expression of costimulatory molecules is likely to impede T cell activation and immunity against SARS-CoV-2 as observed for other viral infections such as influenza virus [68]. Aging-related dysfunction of DCs may be particularly relevant because of DCs role in early control of viral replication and directly affect other innate and adaptive immune subsets.

After establishing the effect of age on myeloid innate immune cells, we turned our attention to NK cells. Taking the role of IFN-I in activating and defining NK cells phenotype into account, we speculated that impaired pDC responses in the elderly patients would result in reduced NK cell responses. Indeed, we found that >65 years patients had reduced percentage of cytokine-producing NK cells (CD56^bright^ NK cells). Exploring their functional characteristics, we found reduced NK cell maturation, differentiation, and functional markers as characterized by reduced CD11b (maturation) and reduced CD122 (differentiation and function) expression in >65 years compared to <65 years patients. Decreased CD122 (IL-2 receptor) expression was accompanied by reduced plasma IL-2 levels in >65 years patients. This dysfunction in the IL-2/CD122 axis may have prognostic implications as indicated by a recent study that suggested that reduced IL-2 levels is a warning factor for disease progression in COVID-19 patients [69]. Together, our findings supported a detrimental impact of aging on NK cell activation and function in COVID-19.

A robust SARS-CoV-2-specific T cell response is critical for clearing the infection and potentially for risk of reinfection as well [26,60]. Effective activation and priming of T cells is mediated by innate immune cells. Taking the extensive dysregulation of innate immune cells in the early symptomatic phase among our older COVID-19 patients into account, we anticipated that the T cell response might also be impacted in these patients. Interestingly, we found reduced activation on effector and memory CD8 T cells among >65 years patients. Plasma IFN-γ levels that were reported to correlate with effective T cells activation [70] were also reduced in the older patients. Decreased expression of T cell costimulatory molecules such as CD86 on all the innate antigen-presenting cells is also a likely contributor to reduced early engagement of T cells. Another reported immune invasion strategy of SARS-CoV-2 is the induction of early T cell exhaustion [71]. Kusnadi et al. reported that severely ill patients displayed increased CD8 T cell exhaustion [72]. Interestingly, our results showed increased CD8 T cell exhaustion (PD-1 expression) among >65 years compared to <65 years patients. Together, our results showed an abrogated early T cell response against SARS-CoV-2 among older COVID-19 patients.

In summary, our study provided evidence that impaired innate cellular responses during the early phase of infection is an underlying pathophysiological mechanism for lack of virological control and higher risk of disease progression in COVID-19 among elderly patients. Thus, our findings provide an important contribution on the current knowledge on the spread of SARS-CoV-2 and COVID-19 pathogenesis.

## Figures and Tables

**Table 1:** Baseline characteristics.

Characteristics	<65 Years of Age (*n* = 116)	≥65 Years of Age (*n* = 89)	*p*-Value
Median age (IQR)—years	53 (46–59)	75 (71–80)	<0.005
Male sex—no. (%)	65 (56)	58 (65)	0.20
Median time (IQR) from symptom onset to baseline—days	9.0 (6.0–12.0)	7.0 (4.0–11.0)	0.01
Median weight (IQR)—kg ^¥^	90 (76–100)	83 (72–92)	0.01
Median body–mass index (IQR)—kg/m^2^ ¤	28.3 (26.1–32.7)	26.6 (23.3–31.4)	0.01
Obesity—no. (%) ^§^	44 (38)	23 (26)	0.07
Symptoms—no. (%)			
Cough	102 (88)	72 (81)	0.17
Dyspnea	86 (74)	51 (57)	0.02
Fatigue	105 (91)	76 (85)	0.28
Headache	75 (65)	32 (36)	<0.005
Coexisting conditions—no. (%)			
Asthma	17 (15)	10 (11)	0.54
COPD	6 (05)	15 (17)	<0.005
Coronary heart disease	10 (09)	29 (33)	<0.005
Hypertension	27 (23)	44 (49)	<0.005
Malignancy	4 (03)	25 (28)	<0.005
Type 2 diabetes	15 (13)	20 (22)	0.09
Score on 7–point ordinal scale—no. (%)			
3. Hospitalized, not requiring supplemental oxygen, requiring ongoing medical care	48 (41)	21 (24)	0.03
4. Hospitalized, requiring supplemental oxygen	60 (52)	60 (67)	
5. Hospitalized, requiring high-flow oxygen therapy or noninvasive ventilation National Early Warning Score 2—median (IQR)	8 (07) 4 (2–6)	8 (09) 5 (3–6)	0.25

IQR denotes interquartile range, and COPD denotes chronic obstructive pulmonary disease. ^¥^ Data on weight was missing for three patients in the <65 years of age group; ¤ Data on body–mass index were missing for four patients in the <65 years of age group and for two patients in the ≥65 years of age group; ^§^ Obesity is defined as a body–mass index of greater than 30.

## Data Availability

Individual participant data cannot be made available due to EU Data Protection Regulations (GDPR). A limited and completely anonymized version of the dataset can be obtained upon request. Study protocols, including laboratory protocols will be available upon request. Proposals should be directed to olesoega@rm.dk.

## Supplementary Materials

The following are available online at https://www.mdpi.com/article/10.3390/jcm10204798/s1, Figure S1: (a) Flow cytometry gating strategy of monocytes. (b) Percentage of monocytes from total PBMC. Mean fluorescence intensity (MFI) of (c) CD169 and (d) CD86 expression in intermediate and nonclassical monocytes. (e) Serum IP-10 level. The data are shown as mean ± SEM. The statistical comparison of below 65 years vs. above 65 years was performed using Student’s *t*-test (* *p* <0.05, ** *p* < 0.01, *** *p* < 0.001, **** *p* < 0.0001). Figure S2: Flow cytometry gating strategy of (a) pDC and mDC and (b) NK cells. Figure S3: Flow cytometry gating strategy of (a) CD8 and CD4 T cells. Percentage of naïve, terminally differentiated, effector, and memory (b) CD8 T cells and (c) CD4 T cells. (d) Serum TNF-α level. The data are shown as mean ± SEM. The statistical comparison of below 65 years vs. above 65 years was performed using Student’s *t*-test (* *p* < 0.05, ** *p* < 0.01, *** *p* < 0.001, **** *p* < 0.0001). Table S1. SARS-CoV-2 Lineages. Table S2. List of antibodies, companies, and identifiers.
