# Supplementary material for: Effect of Age on Innate and Adaptive Immunity in Hospitalized COVID-19 Patients
Source: J Clin Med. 2021 Oct 19;10(20):4798. doi: 10.3390/jcm10204798 (PMC8538457; doi:10.3390/jcm10204798)
Supplement: Supplementary file 1 [file jcm-10-04798-s001.zip › jcm-1413095-supplemenatry materials_figures.pptx]

## Slide 1
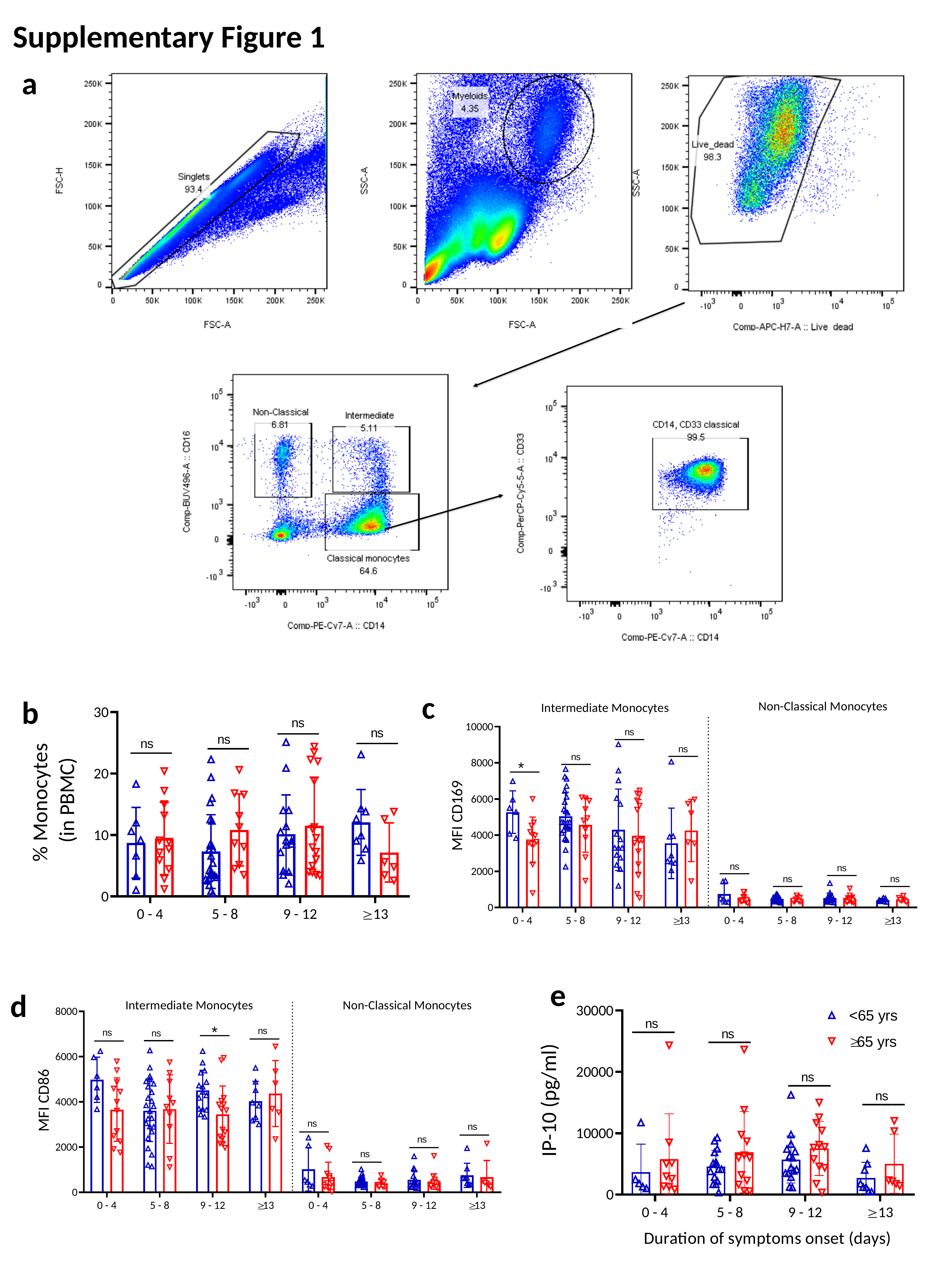

Supplementary Figure 1
a
c
b
e
d

## Slide 2
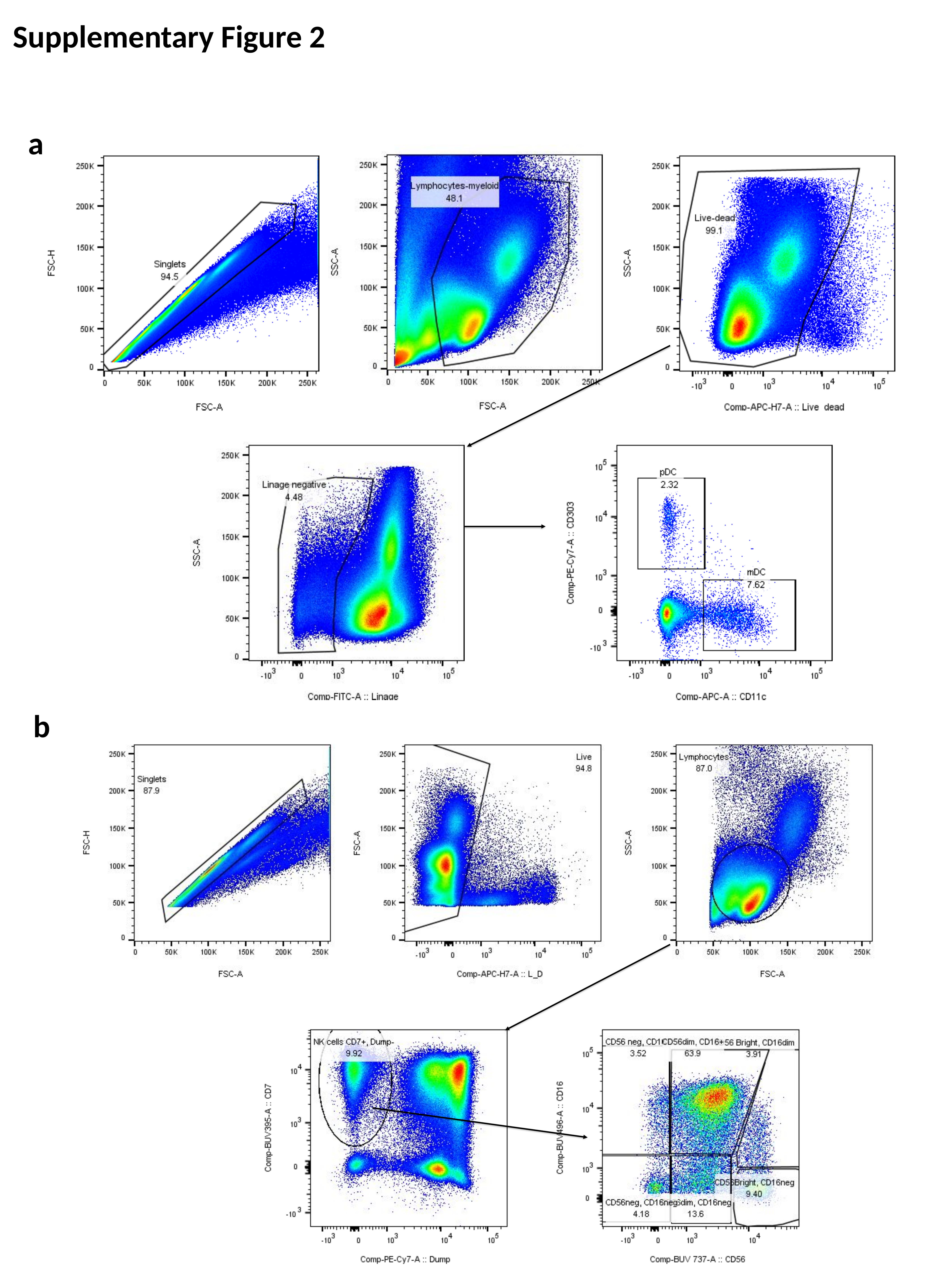

Supplementary Figure 2
a
b

## Slide 3
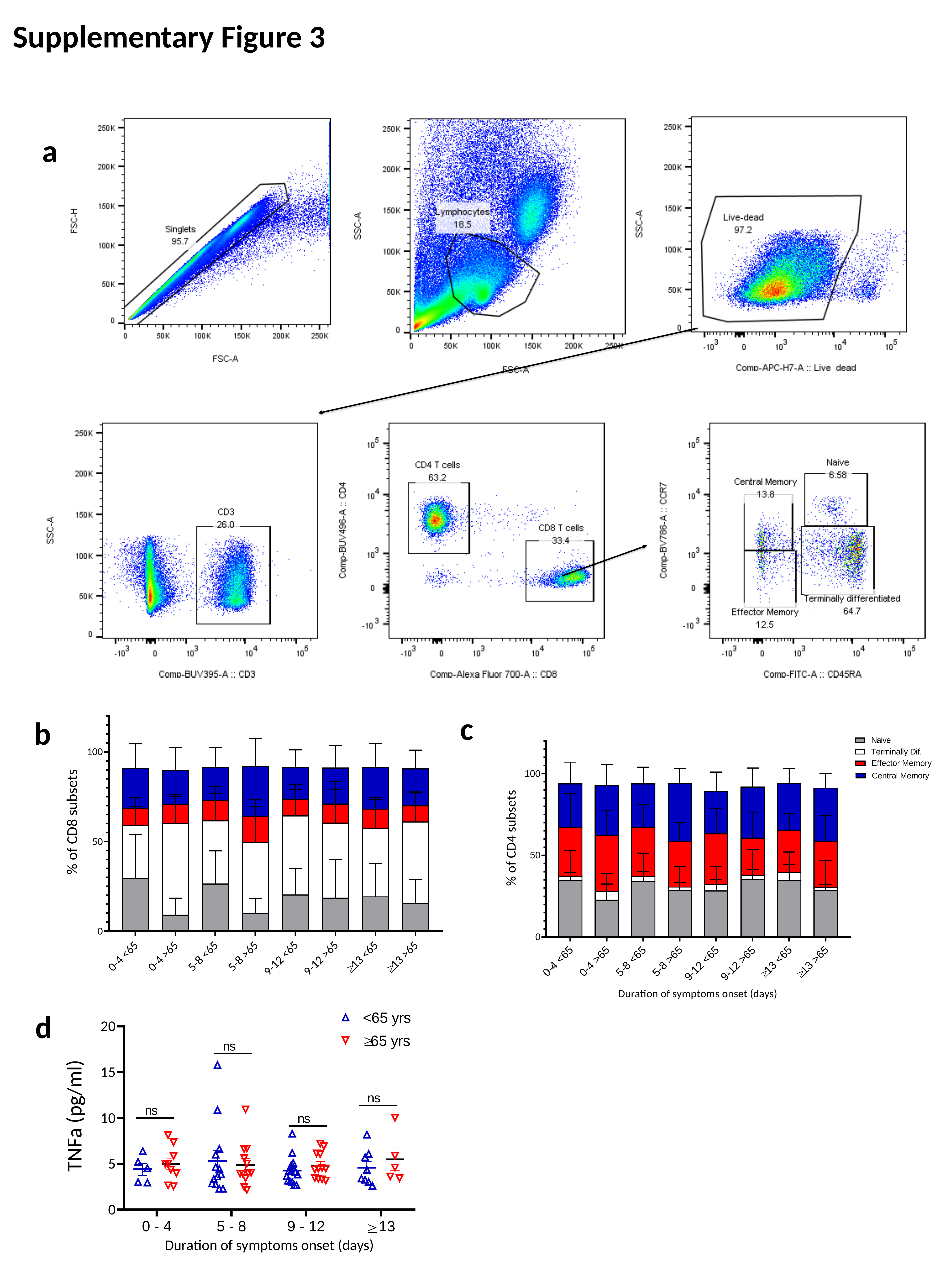

Supplementary Figure 3
a
c
b
d
