# Supplementary material for: Effect of Age on Innate and Adaptive Immunity in Hospitalized COVID-19 Patients
Source: J Clin Med. 2021 Oct 19;10(20):4798. doi: 10.3390/jcm10204798 (PMC8538457; doi:10.3390/jcm10204798)
Supplement: Supplementary file 1 [file jcm-10-04798-s001.zip › jcm-1413095-supplemenatry materials_tables.pdf]

Journal of
Clinical Medicine

MDPI

Table S1. SARS-CoV-2 Lineages.

SARS-CoV-2 Linages	<65 years (n = 50)	>65 years (n = 51)
B.1	8 (16%)	8 (15.6%)
B.1.160	2 (4%)	6 (11.7%)
B.1.258	3 (6%)	7 (13.7%)
B.1.1.288	6 (12%)	4 (7.8%)
B.1.343	5 (10%)	3 (5.8%)
B.1.1.7	1 (2%)	1 (1.9%)
B.1.177	25 (50%)	14 (27.4 %)
B.1.179	0 (0%)	8 (15.6%)

Table S2. List of antibodies, companies, and identifiers.

Antibodies	SOURCE	IDENTIFIER
Anti-CD14	BD Biosciences	*Cat: 557742: RRID: AB_396848*
Anti-CCR2	Biolegend	*Cat: 357208: RRID: AB_2562239*
Anti-CD47	Biolegend	*Cat: 323126: RRID:* AB_2716205
Fixable Near IR	Thermo Fisher	*Cat: L10119: RRID:*
Anti-CD169	BD Biosciences	Cat: 750364*: RRID:* AB_2874539
Anti-CD40	Biolegend	Cat: 334332*: RRID:* AB_2564211
Anti-Lin Cocktail	Biolegend	Cat: 348801: *RRID:* AB_10612570
Anti-CD303	Thermo Fisher	Cat: 25-9818-42*: RRID:* AB_2573552
Anti-CD11c	TonboBio	Cat: 20-0116*: RRID:* AB_2621558
Anti-CD7	BD Biosciences	Cat: 563845*: RRID:* AB_2738448
Anti-CD56	BD Biosciences	Cat: 612766*: RRID:* AB_2813880
Anti-CD122	BD Biosciences	Cat: 562887*: RRID:* AB_2737866
Anti-CD11b	BD Biosciences	Cat: 562721*: RRID:* AB_2737745
Anti-CD161	Biolegend	Cat: 339930*: RRID:* AB_2563968
Anti-CD57	Biolegend	Cat: 359622*: RRID:* AB_2565930
Anti-NKG2A	Beckman Coulter	Cat: IM3291U*: RRID:* AB_10643228
Anti-CD3 (NK only)	BD Biosciences	Cat: 557851*: RRID:* AB_557851
Anti-CD4 (NK only)	BD Biosciences	Cat: 557852*: RRID:* AB_396897
Anti-CD14	BD Biosciences	Cat: BD557742*: RRID:* AB_396848
Anti-CD19	BD Biosciences	Cat: BD557835*: RRID:* AB_396893
Anti-Siglec7	Biolegend	Cat: BL339206*: RRID:* AB_2565239
Anti-CD3	BD Biosciences	Cat:BD564001*: RRID:* AB_2744382
Anti-CD4	BD Biosciences	Cat:BD612936*: RRID:* AB_2870220
Anti-CD38	BD Biosciences	Cat:BD562444*: RRID:* AB_11151894
Anti-PD1	BD Biosciences	Cat:BD563245*: RRID:* AB_2738091
Anti-CD197 (CCR7)	Biolegend	Cat:353230*: RRID:* AB_2563630
Anti-CD45RA	BD Biosciences	Cat:BD555488*: RRID:* AB_395879
Anti-CD69	Biolegend	Cat:BL310910*: RRID:* AB_314845
Anti-CD8	BD Biosciences	Cat:BD565192*: RRID:* AB_2739104
